# Coffee Consumption and the Risk of Thyroid Cancer: A Systematic Review and Meta-Analysis

**DOI:** 10.3390/ijerph14020129

**Published:** 2017-01-27

**Authors:** Mi Ah Han, Jin Hwa Kim

**Affiliations:** 1 Department of Preventive Medicine, College of Medicine, Chosun University, Gwangju 61452, Korea; 2 Department of Internal Medicine, College of Medicine, Chosun University, Gwangju 61452, Korea; endocrine@chosun.ac.kr

**Keywords:** case-control studies, coffee, cohort studies, meta-analysis, review, thyroid neoplasms

## Abstract

An inverse association has been reported between coffee consumption and the risk of several cancers. However, the association between coffee and thyroid cancer is controversial. Thus, this study aimed to evaluate the association between coffee consumption and the risk of thyroid cancer through a systematic review and meta-analysis. Published studies were examined from PubMed, Embase, Cochrane Central, and the reference lists of the retrieved articles. The summary odds ratio (OR) for the association between coffee consumption was categorized as highest versus lowest consumption, and thyroid cancer risk was calculated using a fixed effects model. Subgroup analyses by study design, geographic location, source of controls, and adjusted variables were performed. A total of 1039 thyroid cancer cases and 220,816 controls were identified from five case-control studies and two cohort studies. The summary OR for the association between coffee consumption and thyroid cancer risk was 0.88 (95% confidence interval (CI) = 0.71–1.07). There was no significant heterogeneity among the study results (I² = 0%, *p* = 0.79). However, the beneficial effect of coffee consumption on thyroid cancer was found only in hospital-based case-control studies (OR= 0.59, 95% CI= 0.37–0.93). There was no significant association between coffee consumption and thyroid cancer risk according to our meta-analysis results. These findings should be interpreted with caution because of potential biases and confounding variables. Further prospective studies with a larger number of cases are encouraged to confirm these results.

## 1. Introduction

Thyroid cancer is the eighth and 18th most common cancer among women and men, respectively, in the world [1]. The incidence of thyroid cancer is rapidly increasing, especially among women [2]. Several risk factors for thyroid cancer have been reported, including iodine deficiency or excess, radiation exposure, and female sex [2,3,4].

Coffee is one of the most widely consumed beverages in the world [5]. Coffee contains numerous compounds including caffeine, polyphenols, melanoidins and diterpenes, which have been confirmed to eliminate several carcinogens and reduce the harmful oxidation process [6,7,8]. Many compounds in coffee have the potential to induce biological effects, including antiproliferative, antiangiogenic, and antimetastatic effects [9]. Potential mechanisms for the chemopreventive effects of coffee phytochemicals include inhibition of oxidative stress and oxidative damage as well as regulation of DNA repair, phase II enzymatic activity, apoptosis, and inflammation. Previous epidemiologic studies have evaluated the association between increased coffee consumption and reduced cancer risk. Also, several recent meta-analyses show a positive effect of coffee consumption on preventing liver cancer [10], endometrial cancer [11], and oral cancer [7].

Although many studies have analyzed coffee consumption and its effects on cancers, studies assessing the association between coffee consumption and thyroid cancer risk have yielded inconsistent results. For instance, a case-control study including 284 thyroid cancer cases showed that thyroid cancer patients drank less coffee than healthy patients [12]. However, a pooled analysis of 14 case-control studies from 1980 to 1997 including 2725 thyroid cancer cases reported that there were no clear trends in thyroid cancer risk by level of coffee consumption (a summary odds ratio, OR = 0.9, 95% confidence interval, CI = 0.8–1.1) [13]. A recent meta-analysis to assess the relationship between tea consumption and thyroid cancer risk indicated that higher tea consumption may have a protective effect on thyroid cancer [14].

To our knowledge, a meta-analysis of recently published data has not been performed. Thus, in this study, we performed a meta-analysis to determine the association between coffee consumption and the risk of thyroid cancer. We investigated the risk of thyroid cancer for the highest coffee consumption compared with the lowest consumption.

## 2. Materials and Methods

### 2.1. Data Sources and Search Strategy

A comprehensive systematic search was performed for relevant studies up to 24 June 2016 in PubMed, Embase, and Cochrane Central. Medical Subject Headings, Emtree headings, and other relevant keywords were used to perform the literature search. The keywords used for the search were (caffeine OR coffee) AND (thyroid) AND (tumor OR tumor OR cancer OR neoplasms). In addition, the references from relevant research and review articles were screened to identify additional studies. This review was conducted in line with the preferred reporting items for systematic reviews and meta-analysis (PRISMA) statement [15].

### 2.2. Study Selection and Data Extraction

Two investigators (Mi Ah Han and Jin Hwa Kim) independently reviewed the studies. If there was disagreement, consensus was obtained by discussion. The titles and abstracts were reviewed to identify potentially relevant studies. Full-text manuscripts were reviewed if they met all the following inclusion criteria: (a) studies were based on humans; (b) information on coffee consumption was provided; (c) studies were focused on thyroid cancer; (d) information on the association between coffee consumption and thyroid cancer was provided, including estimates of or calculable information for the OR, risk ratio, or hazard ratio and their corresponding CI; and (e) full-length papers in English since 1990. Reviews and pooled analyses articles were excluded. Since 1990 the coffee market has been subject to the free market forces of supply and demand [16] and then the conditions of coffee consumption were changed substantially [17]. And studies published before 1990 were excluded to reduce excessive diversity in study methods and to minimize study heterogeneity.

Two reviewers (Mi Ah Han and Jin Hwa Kim) used a predesigned data abstraction form to independently collect data from eligible studies. From the selected studies, the following data were extracted: first author’s last name, publication year, country, study design, number of cases, age range, gender, coffee type (caffeinated versus decaffeinated), coffee consumption frequency, type of thyroid cancer, study-specific estimates, and adjusted variables.

### 2.3. Statistical Analyses

An overall summary OR and its 95% CI for highest coffee consumption compared with lowest (i.e., occasional or no) consumption was estimated based on the individual estimates from each study and compared across studies using a forest plot. Each study estimate was given a weight based on the inverse of the variance of the effect estimate. A fixed effect model was used to summarize the study-specific estimates. However, a random effects model was used if there was heterogeneity among study results, which was considered as an I^2^ statistic above 50%. In addition, several subgroup analyses were performed to assess the robustness of the summary estimates. The subgroups were analyzed by study design, geographic location, source of controls, and adjusted variables. Publication bias was not assessed as there were inadequate numbers of included studies to properly assess a funnel plot. Statistical analyses were performed using Reviewer Manager version 5.3 (The Nordic Cochrane Center, Copenhagen, Denmark). Statistical significance was defined as a *p*-value < 0.05.

## 3. Results

### 3.1. Study Selection and Characteristics

**Figure 1:**
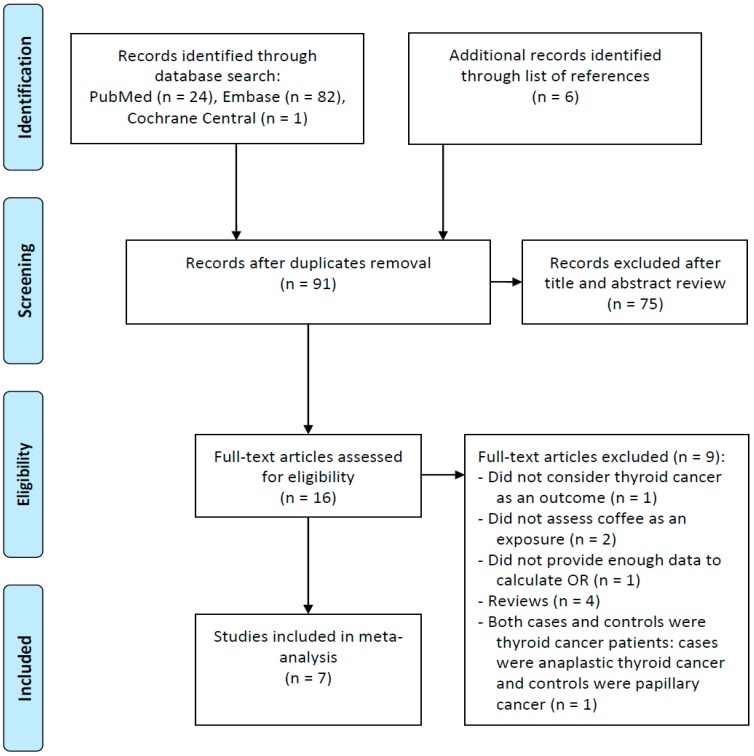
Flowchart of study selection process.

A total of 107 articles published before 24 June 2016 were identified from PubMed, Embase, and Cochrane Central. Six additional articles were retrieved from the reference lists of the identified publications. From these 113 articles, 22 duplicates were removed. The titles and abstracts of the remaining 91 articles were reviewed for relevance. After screening, 16 articles were selected and assessed for eligibility. From these 16 articles, nine were excluded due to the following reasons: one study did not consider thyroid cancer as an outcome, two studies did not assess coffee as an exposure, one study did not provide calculable information for the OR, four studies were review articles, and one case-control study involved thyroid cancer patients for both the case group (anaplastic thyroid cancer) and the control group (papillary cancer). Therefore, seven articles were included in the meta-analysis [18,19,20,21,22,23,24] (Figure 1).

A total of 221,855 participants (1039 thyroid cancer cases) from the seven studies (two cohort studies and five case-control studies) were included in the meta-analysis. From the seven studies, two studies were conducted in the USA, one in Greece, two in Japan, one in Germany, and one in Norway and Sweden. Three studies provided information on caffeinated coffee intake, and five studies provided adjusted risk estimates (Table 1).

### 3.2. Highest Versus Lowest Consumption Meta-Analysis

**Figure 2:**
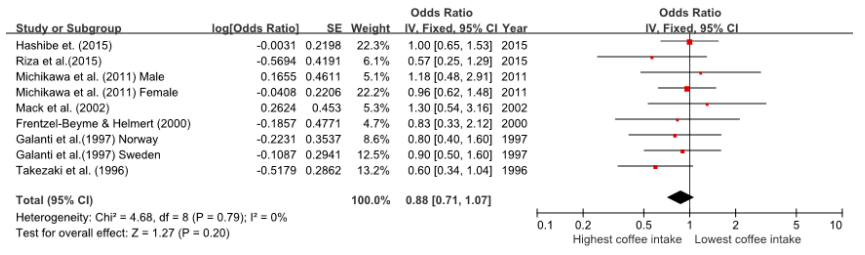
Forest plot of effect estimates from studies on coffee consumption and thyroid cancer risk.

Nine comparisons from the seven studies showed a link between coffee consumption and thyroid cancer risk. The summary OR for the association between thyroid cancer risk and highest versus lowest coffee consumption was 0.88 (95% CI = 0.71–1.07). In addition, the heterogeneity among the included studies was not statistically significant (*p* = 0.79, I^2^ = 0%) (Figure 2).

### 3.3. Subgroup Analyses

No subgroup analyses showed significant heterogeneity among the study results (*p* ≥ 0.05). In the subgroup analysis by the source of controls, an association between coffee consumption and a reduced risk of thyroid cancer was found only in the hospital-based case-control studies (OR = 0.59, 95% CI = 0.37–0.93) but not in the population-based case-control studies (Table 2).

## 4. Discussion

Our meta-analysis included two prospective cohort studies, three population-based case-control studies, and two hospital-based case-control studies. The analysis was performed to assess the effects of coffee consumption on the risk of thyroid cancer. Based on seven published studies including 1039 thyroid cancer cases, the summary OR for the risk of thyroid cancer with the highest coffee consumption compared with no/occasional consumption was 0.88 (95% CI = 0.71–1.07). Among hospital-based case-control studies, the summary OR was 0.59 (95% CI = 0.37–0.93). However, hospital-based controls might alter their coffee consumption behavior according to their medical condition [25]. When considering the coffee is associated with health, hospital-based controls might overstate their actual coffee consumption more than population-based controls [26].

Coffee contains a variety of biologically active compounds that might affect the risk of cancer. For instance, caffeine increases intracellular cyclic adenosine monophosphate, which has an inhibitory effect on cell (i.e., tumor) growth [13,27]. However, the effects of caffeinated versus decaffeinated coffee on thyroid cancer were not studied in this meta-analysis due to limited information. Only one study [22] reported separate association estimates for caffeinated and decaffeinated coffee with thyroid cancer, showing that drinking caffeinated coffee has a preventive effect on thyroid cancer, whereas decaffeinated coffee was associated with an increased risk [22]. Moreover, animal and cell culture studies have suggested that additional active compounds in coffee, such as cafestol and kahweol, have antioxidant and antimutagenic properties and might activate enzymes related to carcinogenic detoxification [7]. Inverse dose-response relationships were founded between coffee consumption and cancer risk [11,28].

Most study-specific estimates included in this meta-analysis were adjusted for several risk factors for thyroid cancer such as age [19,20,24], sex [19] and prior benign thyroid disease [21,24]. However, several studies did not provide adjusted estimates. The unavailability of these data prevented us from performing a meta-analysis using specific strata of covariates. Therefore, we cannot not exclude some residual confounding variables, such as prior benign thyroid disease and iodine intake, for which several original studies were not adjusted. Caffeine, the best-known component of coffee, has different effects on thyroid cancer risk depending on levels of iodine intake [29]. Because most studies in this meta-analysis did not provide information on iodine intake, we were unable to associate this risk factor with the effect of coffee on thyroid cancer risk. However, a previous study reported differences in iodine status at the regional and global levels [30]. So, studies that consider iodine intake status are needed to evaluate its effect on the association between coffee and thyroid cancer risk. Another consideration regarding these study results is coffee consumption behavior. The amount and type (i.e., caffeinated versus decaffeinated) of consumed coffee differ considerably between various locations [31,32,33]. Notably, coffee consumption is especially high in Europe and relatively low in Asia [34]. In addition, the composition of coffee and its effect on health may differ depending on the method of preparation (i.e., drip-filtered vs. boiled vs. instant coffee) [35,36].

The coffee consumption rate, awareness about the components of coffee and its effect on health and body were significantly different by gender [37]. This difference can affect the composition and concentration of the coffee compound and its health effect in men and women. In previous meta-analysis which investigated the association between coffee consumption and several types of cancer, there were no significant differences by sex [38]. On the other hand, the association or statistical significance was different by sex [6,26]. In our study, we did not conduct a stratified analysis by sex due to limited information and most included studies did not calculate study-specific estimates after controlling for gender. Further study should reveal the effect of gender on association between coffee consumption and thyroid cancer risk.

There are limitations of this meta-analysis that call for caution when interpreting the results. First, the majority of included studies were retrospective, whereas only two studies were prospective cohort studies. Retrospective studies are prone to recall bias [39]. Although there was no evidence of significant heterogeneity among studies, the summary estimates differed by study design in the subgroup analysis. It is possible that because a relatively small number of cohort studies were included, the results of case-control studies may have been overstated as a result of recall or interviewer bias. Further prospective studies are needed to elucidate the association between coffee and thyroid cancer risk. Second, because of inconsistent categorization in coffee consumption across studies, we did not perform a subgroup analysis based on coffee consumption behavior or calculate a dose-response relationship. Instead, we only calculated pooled estimates for the highest coffee consumption versus the lowest coffee consumption. Third, the included studies did not describe the concentrations of caffeine, methods of coffee preparation, serving size, or type of coffee beans, which may have biased our results [40]. Fourth, the risk of thyroid cancer by histological type was not assessed due to limited data. Finally, a test for funnel plot asymmetry was not conducted because when there are fewer than 10 studies, it is difficult to identify for publication bias using the funnel plot [41].

To our knowledge, this is the first meta-analysis to evaluate the association between coffee consumption and thyroid cancer risk. Our meta-analysis includes a large number of thyroid cases, which enables a greater possibility of reaching reasonable conclusions about the link between coffee consumption and thyroid cancer risk.

The results of this meta-analysis suggest that coffee consumption has no effect on the risk of thyroid cancer with the exception hospital-based case-control studies. These findings should be interpreted with caution because of potential biases and confounding variables. Future studies are suggested to assess the difference by gender and histological type of thyroid cancer. Also, prospective studies that consider more detailed coffee consumption behavior (coffee type, amount, preparation methods, etc.) and the dose-response relationship between coffee consumption and thyroid cancer risk are necessary to support our results.

## Figures and Tables

**Table 1:** Characteristics of studies on coffee consumption and thyroid cancer risk.

Author (year)	Country	Study Design	Participants (cases)	Age Range (years)	Gender	Coffee Type	Histologic Type	Definition of Coffee Consumption (Highest vs. Lowest Intake)	OR or RR (95% CI) for Highest vs. Lowest Intake	Adjusted Variables
Hashibe et al. (2015) [19]	USA (North America)	PCS	97,334 (106)	≥55	Male, Female	Caffeinated and decaffeinated	NR	≥2 cups per day vs. <1 cup per day	1.00 (0.65–1.53)	Age, sex, race, and education
Riza et al. (2015) [18]	Greece (Europe)	HCC	251 (113)	Mean Cases: 44.5 Controls: 38.2	Male, Female	NR	PTC:FTC:MTC:O = 78:8:10:4	Yes vs. no	0.57 (0.25–1.29)	None
Michikawa et al. (2011) [20]	Japan (Asia)	PCS	100,507 (159)	40–69	Male, Female	NR	PTC:FTC:MTC:O = 83.6:4.4:0.6:11.4	≥1 cup per day vs. almost never	M: 1.18 (0.48–2.91); W: 0.96 (0.62–1.48)	Age, area, smoking history, passive smoking in the workplace, alcohol consumption, body mass index, consumption of green vegetables and seaweed, health screening in the previous year, green tea consumption, menopausal status, and use of exogenous female hormones. M: Same as above except menopausal status and green tea consumption
Mack et al. (2002) [21]	USA (North America)	PCC	294 (147)	15­–54	Female	Caffeinated	PTC:O = 86.4:13.6	6 or more cups per day vs. none	All thyroid cancers: 1.3 (0.5–3.1). Papillary thyroid cancer: 1.4 (0.6–3.4)	Prior benign thyroid disease
Frentzel-Beyme and Helmert (2000) [22]	Germany (Europe)	PCC	338 (174)	Mean: 51.4	Male, Female	Caffeinated and decaffeinated	PTC:FTC:O = 64.9:25.3:9.8	>5 cups per day vs. never	Caffeinated coffee: 0.83 (0.33–2.12). Decaffeinated coffee: 2.70 (0.72–10.2)	Very high consumption of coffee with caffeine, decaffeinated coffee, black tea, and alcoholic beverages
Galanti et al. (1997) [23]	Norway; Sweden; (Europe)	PCC	686 (246)	18–75	Male, Female	NR	PTC:FTC = 85:15	>150 vs. ≤89 portions per month	Norway: 0.8 (0.4–1.6). Sweden: 0.9 (0.5–1.6)	None
Takezaki et al. (1996) [24]	Japan (Asia)	HCC	2,2760 (94)	20–79	Female	NR	PTC:FTC = 97:3	Everyday vs. less	0.6 (0.3–1.04)	Age, year of visit, past history of thyroid disease, Western-style breakfast, and prior parturition

FTC, follicular thyroid cancer; HCC, hospital-based case-control study; M, medullary thyroid cancer; NR, not reported; O, Other types of thyroid cancer; PTC, papillary thyroid cancer; PCC, population-based case-control study; PCS, prospective cohort study; RR, relative risk.

**Table 2:** Subgroup analysis for association between coffee consumption and thyroid cancer risk.

Subgroups	No. of Studies (No. of Comparisons)	OR (95% CI)	Heterogeneity Test (I^2^, *p*-Value)
All studies	7 (9)	0.88 (0.71–1.07)	0%, 0.79
Study design			
Cohort	2 (3)	1.00 (0.75–1.33)	0%, 0.92
Case-control	5 (6)	0.77 (0.58–1.03)	0%, 0.70
Geographic location			
Europe	3 (4)	0.79 (0.55–1.13)	0%, 0.84
North America	2 (2)	1.05 (0.71–1.55)	0%, 0.60
Japan	2 (3)	0.84 (0.61–1.16)	15%, 0.31
Source of controls			
Population-based	3 (4)	0.91 (0.63–1.32)	0%, 0.85
Hospital-based	2 (2)	0.59 (0.37–0.93)	0%, 0.92
Adjustment			
Yes	5 (6)	0.92 (0.72–1.16)	0%, 0.64
No	2 (3)	0.78 (0.53–1.15)	0%, 0.66

CI, confidence interval; OR, odds ratio.

## References

[1] Torre L.A., Bray F., Siegel R.L., Ferlay J., Lortet-Tieulent J., Jemal A. (2015). Global cancer statistics, 2012. CA Cancer J. Clin..

[2] Davies L., Welch H.G. (2014). Current thyroid cancer trends in the United States. JAMA Otolaryngol. Head Neck Surg..

[3] Shakhtarin V.V., Tsyb A.F., Stepanenko V.F., Orlov M.Y., Kopecky K.J., Davis S. (2003). Iodine deficiency, radiation dose, and the risk of thyroid cancer among children and adolescents in the Bryansk region of Russia following the Chernobyl power station accident. Int. J. Epidemiol..

[4] Zimmermann M.B., Galetti V. (2015). Iodine intake as a risk factor for thyroid cancer: A comprehensive review of animal and human studies. Thyroid Res..

[5] Cornelis M.C. (2015). Toward systems epidemiology of coffee and health. Curr. Opin. Lipidol..

[6] Li G., Ma D., Zhang Y., Zheng W., Wang P. (2015). Coffee consumption and risk of colorectal cancer: A meta-analysis of observational studies. Public Health Nutr..

[7] Li Y.M., Peng J., Li L.Z. (2016). Coffee consumption associated with reduced risk of oral cancer: A meta-analysis. Oral Surg. Oral Med. Oral Pathol. Oral Radiol..

[8] Wierzejska R. (2015). Coffee consumption vs. cancer risk—A review of scientific data. Rocz. Państw. Zakł. Hig..

[9] Bohn S.K., Blomhoff R., Paur I. (2014). Coffee and cancer risk, epidemiological evidence, and molecular mechanisms. Mol. Nutr. Food Res..

[10] Bravi F., Tavani A., Bosetti C., Boffetta P., La Vecchia C. (2016). Coffee and the risk of hepatocellular carcinoma and chronic liver disease: A systematic review and meta-analysis of prospective studies. Eur. J. Cancer Prev..

[11] Zhou Q., Luo M.L., Li H., Li M., Zhou J.G. (2015). Coffee consumption and risk of endometrial cancer: A dose-response meta-analysis of prospective cohort studies. Sci. Rep..

[12] Przybylik-Mazurek E., Hubalewska-Dydejczyk A., Kuzniarz-Rymarz S., Kiec-Klimczak M., Skalniak A., Sowa-Staszczak A., Golkowski F., Kostecka-Matyja M., Pach D. (2012). Dietary patterns as risk factors of differentiated thyroid carcinoma. Postepy Hig. Med. Dosw..

[13] Mack W.J., Preston-Martin S., Dal Maso L., Galanti R., Xiang M., Franceschi S., Hallquist A., Jin F., Kolonel L., La Vecchia C. (2003). A pooled analysis of case-control studies of thyroid cancer: Cigarette smoking and consumption of alcohol, coffee, and tea. Cancer Causes Control.

[14] Ma S., Wang C., Bai J., Wang X., Li C. (2015). Association of tea consumption and the risk of thyroid cancer: A meta-analysis. Int. J. Clin. Exp. Med..

[15] Moher D., Liberati A., Tetzlaff J., Altman D.G., PRISMA Group (2009). Preferred reporting items for systematic reviews and meta-analyses: The PRISMA statement. PLoS Med..

[16] International Cancer Organization (2014). World coffee trade (1963–2013): A Review of the Markets, Challenges and Opportunities Facing the Sector.

[17] Ponte S. (2002). The “latte revolution”? Regulation, markets and consumption in the global coffee chain. World Dev..

[18] Riza E., Linos A., Petralias A., de Martinis L., Duntas L., Linos D. (2015). The effect of Greek herbal tea consumption on thyroid cancer: A case-control study. Eur. J. Public Health.

[19] Hashibe M., Galeone C., Buys S.S., Gren L., Boffetta P., Zhang Z.F., La Vecchia C. (2015). Coffee, tea, caffeine intake, and the risk of cancer in the PLCO cohort. Br. J. Cancer.

[20] Michikawa T., Inoue M., Shimazu T., Sasazuki S., Iwasaki M., Sawada N., Yamaji T., Tsugane S. (2011). Green tea and coffee consumption and its association with thyroid cancer risk: A population-based cohort study in Japan. Cancer Causes Control.

[21] Mack W.J., Preston-Martin S., Bernstein L., Qian D. (2002). Lifestyle and other risk factors for thyroid cancer in Los Angeles County females. Ann. Epidemiol..

[22] Frentzel-Beyme R., Helmert U. (2000). Association between malignant tumors of the thyroid gland and exposure to environmental protective and risk factors. Rev. Environ. Health.

[23] Galanti M.R., Hansson L., Bergstrom R., Wolk A., Hjartaker A., Lund E., Grimelius L., Ekbom A. (1997). Diet and the risk of papillary and follicular thyroid carcinoma: A population-based case-control study in Sweden and Norway. Cancer Causes Control.

[24] Takezaki T., Hirose K., Inoue M., Hamajima N., Kuroishi T., Nakamura S., Koshikawa T., Matsuura H., Tajima K. (1996). Risk factors of thyroid cancer among women in Tokai, Japan. J. Epidemiol..

[25] Silverman D.T., Hoover R.N., Swanson G.M., Hartge P. (1983). The prevalence of coffee drinking among hospitalized and population-based control groups. J. Am. Med. Assoc..

[26] Wu W., Tong Y., Zhao Q., Yu G., Wei X., Lu Q. (2015). Coffee consumption and bladder cancer: A meta-analysis of observational studies. Sci. Rep..

[27] Linos A., Linos D.A., Vgotza N., Souvatzoglou A., Koutras D.A. (1989). Does coffee consumption protect against thyroid disease?. Acta Chir. Scand..

[28] Yu C., Cao Q., Chen P., Yang S., Deng M., Wang Y., Li L. (2016). An updated dose-response meta-analysis of coffee consumption and liver cancer risk. Sci. Rep..

[29] Son H.Y., Nishikawa A., Kanki K., Okazaki K., Kitamura Y., Lee K.Y., Umemura T., Hirose M. (2003). Synergistic interaction between excess caffeine and deficient iodine on the promotion of thyroid carcinogenesis in rats pretreated with *N*-bis(2-hydroxypropyl)nitrosamine. Cancer Sci..

[30] Andersson M., Karumbunathan V., Zimmermann M.B. (2012). Global iodine status in 2011 and trends over the past decade. J. Nutr..

[31] Grosso G., Stepaniak U., Micek A., Stefler D., Bobak M., Pajak A. (2016). Coffee consumption and mortality in three Eastern European countries: Results from the HAPIEE (Health, Alcohol and Psychosocial factors In Eastern Europe) study. Public Health Nutr..

[32] Makiuchi T., Sobue T., Kitamura T., Ishihara J., Sawada N., Iwasaki M., Sasazuki S., Yamaji T., Shimazu T., Tsugane S. (2016). Association between green tea/coffee consumption and biliary tract cancer: A population-based cohort study in Japan. Cancer Sci..

[33] Gloess A.N., Schönbächler B., Klopprogge B., Lucio D., Chatelain K., Bongartz A., Strittmatter A., Rast M., Yeretzian C. (2013). Comparison of nine common coffee extraction methods: Instrumental and sensory analysis. Eur. Food Res. Technol..

[34] Je Y., Giovannucci E. (2014). Coffee consumption and total mortality: A meta-analysis of twenty prospective cohort studies. Br. J. Nutr..

[35] Nilsson L.M., Wennberg M., Lindahl B., Eliasson M., Jansson J.H., van Guelpen B. (2010). Consumption of filtered and boiled coffee and the risk of first acute myocardial infarction; a nested case/referent study. Nutr. Metab. Cardiovasc. Dis..

[36] Ranheim T., Halvorsen B. (2005). Coffee consumption and human health-beneficial or detrimental?—Mechanisms for effects of coffee consumption on different risk factors for cardiovascular disease and type 2 diabetes mellitus. Mol. Nutr. Food Res..

[37] Demura S., Aoki H., Mizusawa T., Soukura K., Noda M., Sato T. (2013). Gender differences in coffee consumption and its effects in young people. Food Nutr. Sci..

[38] Yu X., Bao Z., Zou J., Dong J. (2011). Coffee consumption and risk of cancers: A meta-analysis of cohort studies. BMC Cancer.

[39] Song J.W., Chung K.C. (2010). Observational studies: Cohort and case-control studies. Plast. Reconstr. Surg..

[40] Bracken M.B., Triche E., Grosso L., Hellenbrand K., Belanger K., Leaderer B.P. (2002). Heterogeneity in assessing self-reports of caffeine exposure: Implications for studies of health effects. Epidemiology.

[41] Sterne J.A., Sutton A.J., Ioannidis J.P., Terrin N., Jones D.R., Lau J., Carpenter J., Rucker G., Harbord R.M., Schmid C.H. (2011). Recommendations for examining and interpreting funnel plot asymmetry in meta-analyses of randomised controlled trials. BMJ.

